# Editorial: Optimizing Local Therapy for High-Risk Prostate Cancer: Evidence and Emerging Options

**DOI:** 10.3389/fonc.2020.01616

**Published:** 2020-08-27

**Authors:** Nicholas G. Zaorsky, Daniel E. Spratt, Amar U. Kishan, Stephen H. Culp, Timothy N. Showalter

**Affiliations:** ^1^Department of Radiation Oncology, Penn State Cancer Institute, Hershey, PA, United States; ^2^Department of Radiation Oncology, Michigan Medicine, University of Michigan, Ann Arbor, MI, United States; ^3^Department of Radiation Oncology, University of California, Los Angeles, Los Angeles, CA, United States; ^4^Department of Urology, University of Virginia, Charlottesville, VA, United States; ^5^Department of Radiation Oncology, School of Medicine, University of Virginia, Charlottesville, VA, United States

**Keywords:** high risk prostate cancer, androgen deprivation therapy, radical prostatecomy, recurrent prostate cancer, radiation therapy

Recent evidence has suggested an important role for local therapy across the spectrum of prostate cancer, including localized and as well as low-volume metastatic prostate cancers, in maximizing cure rates for prostate cancer (1–3). The National Comprehensive Cancer Network (NCCN) guidelines have changed dramatically for these patients in the past several decades. For example, in 2012, for high risk localized disease, the guidelines generally recommended definitive external beam radiation therapy + androgen deprivation therapy (category 1) and radical prostatectomy and appropriate adjuvant or salvage therapy (category 2). In 2017, the guidelines changed so that surgical intervention had a category 1 recommendation; however, in 2019, the guidelines changed again to include external beam radiation therapy + androgen deprivation therapy (category 1) or external beam radiation therapy + brachytherapy boost + androgen deprivation therapy (category 2).

These changes in the guidelines came from newly published studies, and as of 2020, the ideal management of high-risk prostate cancer continues to evolve, mostly because almost all studies have been observational and retrospective (4, 5). A randomized trial of surgery vs. radiation therapy in the setting of high risk disease has only recently gotten underway with the SPCG-15 trial (6), which randomizes between radical prostatectomy vs. androgen deprivation therapy in combination with external beam radiation therapy ± high dose-rate brachytherapy boost.

Further, in patients with low volume metastatic disease, novel therapeutic combination approaches directed toward the primary tumor, and potentially areas of metastasis, are being investigated as strategies to increase cure rates and extend life for men with high risk and metastatic prostate cancers (7–9). Although this is an exciting area for research and contemporary clinical practice for prostate cancer, a range of considerations remain undefined.

This collection features contributions on a range of topics that summarize the best available evidence on this topic and highlight emerging advances that will improve prostate cancer care in the years to come. Several manuscripts focus on the use of laboratory, imaging and pathological information to more accurately predict outcomes after treatment and to tailor therapeutic strategies (Bourbonne et al.; Chys et al.; Guo et al.; Milonas et al.; Venclovas et al.). Motterle et al. review the role of radical prostatectomy for regional risk prostate cancer patients, while Devos et al. investigate the impact of robot-assisted prostatectomy in recurrent prostate cancer. Additionally, three manuscripts consider the impact of radiation technical advances on outcomes for high-risk or node-positive prostate cancer (Fischer-Valuck et al.,Greenberger et al.; Koerber et al.). Harat et al. evaluate the comparative effectiveness and cost effectiveness of local therapy options for localized prostate cancer, providing a comprehensive view of treatment options. Finally, Mao et al. provide a peak into the future role of the novel treatment strategy of oncoloytic adenovirus harboring interleukin 24 in combination with radiation therapy to enhance outcomes for advanced prostate cancer (Mao et al.).

Our hope is that this collection of articles contributes to the ongoing interdisciplinary discussions on this topic to continue to improve outcomes for high risk prostate cancer. We believe that tremendous impact can be realized by improving treatment strategies for men with high-risk prostate cancer, as advances in management of locally advanced, node-positive, and low-burden metastatic disease will translate in reduced recurrence risk for men with high risk of metastasis at time of diagnosis.

## Author Contributions

All authors listed have made a substantial, direct and intellectual contribution to the work, and approved it for publication.

## Conflict of Interest

The authors declare that the research was conducted in the absence of any commercial or financial relationships that could be construed as a potential conflict of interest.
